# The socioeconomic impact of inherited retinal dystrophies (IRDs) in Belgium: A cost-of-illness study

**DOI:** 10.1371/journal.pone.0339332

**Published:** 2026-01-27

**Authors:** Ine Vandersmissen, Janice Geers, Tom Denee, Salla Oinasmaa, Dagmar Hoeben, Xinyi Zhang, Burcak Aydin, Simone Cheung, Lorenzo Billiet, Inge Joniau, Avril Daly, Steven Simoens, Bart P. Leroy

**Affiliations:** 1 Johnson & Johnson, Beerse, Belgium; 2 Johnson & Johnson, Breda, The Netherlands; 3 Johnson & Johnson, Espoo, Finland; 4 Health Economics and Outcomes Research, Monitor Deloitte, Brussels, Belgium; 5 Health Economics and Social Policy, Deloitte Access Economics, Melbourne, Victoria, Australia; 6 Licht en Liefde, Varsenare, Belgium; 7 Department of Ophthalmology, Ghent University Hospital, Ghent, Belgium; 8 Retina International, Dublin, Ireland; 9 Department of Pharmaceutical and Pharmacological Sciences, KU Leuven, Leuven, Belgium; 10 Center for Medical Genetics, Ghent University Hospital, Ghent, Belgium; 11 Department of Head & Skin, Ghent University, Ghent, Belgium; University of Tübingen, GERMANY

## Abstract

**Introduction:**

Inherited retinal diseases (IRDs) are a diverse group of vision-threatening conditions caused by genetic mutations, affecting over 5.5 million people globally. These diseases have profound impact on patients, families and society. However, there is a lack of comprehensive data on their prevalence, economic burden, and societal costs. This information gap hinders effective decision-making, and the allocation of resources needed for research, treatment, and patient support, ultimately compromising care and outcomes.

**Objective:**

To quantify the societal burden of IRDs in Belgium in 2023

**Methods:**

This cost-of-illness study estimated the total costs of the 11 most prevalent IRDs in Belgium. A survey of Belgian IRD patients collected primary data on disease burden, health resource utilization, productivity, and care-related expenses. Additionally, data on prevalence and health system costs were based on a literature review from PubMed and the Cochrane Library.

**Results:**

Patients reported a substantial impact on daily activities (96%), mental health (81%), and 78% reported having received genetic testing. IRDs impose a significant economic burden in Belgium, with an average annual cost of €37,228 per patient and a total cumulative burden of €129.4 million. Indirect non-healthcare costs represented the largest proportion (84%), followed by direct healthcare costs (12%), mainly from rehabilitation, and direct non-healthcare costs (4%). Indirect costs included significant productivity losses of €77.7 million and an informal care burden of €31.3 million. Government support programmes accounted for €4.9 million annually.

**Conclusion:**

This study highlights the substantial clinical and economic burden of IRDs on Belgian society. Therefore, it is important to continue investing in research and development, as well as to consider the substantial societal impact of these conditions in decision-making and shaping policy changes for Belgium. Future investments informed by these findings can contribute to decreasing the burden on society and enhance the well-being, inclusion and productivity of patients and caregivers.

## Introduction

Inherited retinal diseases (IRDs) represent a diverse group of progressive, visually debilitating diseases in which genetic mutations critical to retinal function lead to progressive photoreceptor cell death and loss of retinal pigment epithelium (RPE), which is ultimately associated with vision loss [1,2]. IRDs are the most common cause of blindness of people in the working-age and therefore have a meaningful impact on society [3]. Inheritance patterns identified in IRDs include autosomal dominant, autosomal recessive, X-linked and mitochondrial inheritance [4]. However, some IRDs can also arise through new genetic variants [5]. IRDs are classified as rare diseases due to their low prevalence, and their genetic and clinical heterogeneity adds to the complexity of diagnosis and treatment [6]. Although there are phenotypical commonalities between IRDs, it should be noted that the physical and psychosocial disability of each condition is highly varied, which adds complexity to comparing the degree of disability of each condition.

There currently is only one marketed treatment available: voretigene neparovec for the treatment of *RPE65-*IRD, which was approved by the the European Medicines Agency in 2018 [7]. Several therapies are currently in clinical development to provide therapeutic options that could delay or halt the rate of disease progression, with some success of neurotrophic factor therapy and gene therapies reported from clinical trials [1]. While voretigene neparvovec offers relief to a small subset of *RPE65*-IRD patients, the vast genetic heterogeneity across IRDs means that the large majority of patients still lack effective treatment options. Even with emerging therapies, a significant gap remains, as many types of IRDs are caused by mutations that cannot yet be addressed by available treatments. Therefore, continued efforts are required to develop alternative therapies that can address the diverse genetic causes of IRDs.

Quality of life in patients with IRDs is significantly impacted as the disease progresses and causes significant visual impairment and blindness [8]. People living with visual impairment often live with other comorbidities [9]. One study on patients living with visual impairment reported that their autonomy significantly decreased over time, with 37% reporting complete autonomy at diagnosis and 23% at last visit [10]. In addition to loss of autonomy, IRD patients are more likely to develop depression in comparison to individuals without vision impairment. As IRDs typically develop during childhood, parents of children with vision impairment often cite concerns about the psychosocial impact of their child’s condition [11]. These findings highlight that, in addition to the physical limitations of the patient, vision impairment also imposes a significant clinical impact on the mental health of patients and their families.

Globally, an estimated 1 in 3,000 individuals are affected by an IRD, with no specific data available for prevalence in Belgium [12]. While previous studies have highlighted that the economic and humanistic losses of IRDs are significant, limited if any evidence exists on the specific burden within Belgium, despite its significant impact on the patients and their families.

To date, no comprehensive cost-of-illness analysis has been carried out with a view to quantifying the burden that IRDs impose on society in Belgium. This study therefore aimed to assess the economic burden of IRDs in Belgium in 2023 based on the impact patients and their caregivers experience through primary data collection.

## Materials and methods

### Patient survey

Data on the healthcare resource utilization, out-of-pocket expenses on aids and modifications and treatment, formal and informal care, productivity impact and usage of government support for patients with IRD, were obtained via a survey. The study population comprised IRD patients living in Belgium. Participants who had previously received gene or cell therapy to treat their condition were excluded as the aim of this study is to show the impact of untreated IRDs on society to allow for an unbiased view upon the needs for future investments and potential savings.

Questions in the survey related to (i) sociodemographic factors, (ii) disease characteristics, (iii) healthcare resource utilization, (iv) impact on work situation, and (v) government support programs (S1 Table) and were validated by Belgian clinical & health-economic experts and patient associations (Licht en Liefde and Brailliga). Patients were asked to consider their answers over the past year and the data were, where necessary, extrapolated to cover for a one-year period.

### Patient recruitment

The questionnaire was launched by PIPHealth via an online registration form on the PIPHealth webpage on July 27^th^ 2023 and remained open until October 2^nd^. Patients were informed about the survey through the e-mail database of PIPHealth and a patient association, social media and flyers that were also available through IRD clinics at Ghent University Hospital, the national Belgian specialist center for IRDs. Patients reported the diagnosis of their hereditary retinal disorder previously made by an expert healthcare professional in response to the question “Which hereditary retinal disorder have you been diagnosed with?”, and indicated whether genetic testing had been performed, which had been conducted for 78.2% of respondents. Non-probabilistic convenience sampling was employed to ensure the greatest number of respondents.

The study was reviewed by the Commission for Medical Ethics of University Hospital Ghent. In accordance with the Belgian law of 07/05/2004, the committee (request ONZ-2023–0160) confirmed that the study does not fall within the scope of this legislation and therefore does not require formal ethical committee approval. All survey participants provided written informed consent prior to participation.

Besides being a recruiting partner, PIPHealth was also involved in the set-up of the survey and the anonymization and aggregation of the data, and no patient level identifying information was accessible for parties who were involved in the analysis. The survey data was analyzed using descriptive statistics, including mean, median and range quantifications. Deloitte received the aggregated data from the PIP Health and ran the descriptive statistics which was only accessible to the Deloitte statistician. In accordance with the patient consent form and Belgian data protection regulations, the primary dataset from the patient survey has been provided in S1 Table in an anonymized and aggregated format.

### Epidemiology

A targeted review of the scientific literature was performed using PubMed and Cochrane databases to collect international prevalence data, as no national Belgian prevalence data were available for the IRDs of interest. The distribution of prevalence by age and sex for each condition was derived based on the distributions described in the eyeGENE registry data [13].

### Cost categories

The cost-of-illness study adopts a prevalence-based approach to estimate the economic burden associated with IRDs in Belgium for one given year (2023). The cost components were included in the following categories:

- Direct Healthcare Costs: Expenditures on primary healthcare services, specialty care, hospitalization, medication (limited to supporting medications only), genetic testing, and formal care(e.g., private nursing, housekeeping and personal assistance delivered by trained caregivers or social workers). Treatment costs were included based on the patient reported out-of-pocket expenditures.
- Direct Non-Healthcare Costs: Expenses on aids and modifications. Aids and modifications were included based on the patient reported out-of-pocket expenditures.
- Indirect Non-Healthcare Costs: costs from reduced working participation, absenteeism, presenteeism, and informal care (e.g., support from friends and family for daily activities).
- Government support programs

The expenditure is assessed from the societal perspective, including direct healthcare costs paid for in the context of the compulsory national scheme for health insurance and disability benefits and government support programs at a federal level, as well as regional government funding, including rehabilitation in expert centers and low vision departments as well as regional government support programs, and patients’ copayments and expenses, as depicted in Table 1.

**Table 1:** Cost per category and per payer.

			Patient	Public healthcare payer	Regional Government	Total
Direct healthcare costs	Primary care	General Practitioner	✓	✓		✓
	Specialty care	Retina specialist visit	✓	✓		✓
		Ophthalmologist visit	✓	✓		✓
		Psychologist or psychiatric visit	✓	✓		✓
		Physiotherapist or kinesist visit	✓	✓		✓
	Rehabilitation	Rehabilitation center or center of expertise	✓		✓	✓
		Low vision department	✓		✓	✓
		Counselor professional organization	✓		✓	✓
	Hospitalization	Hospitalization	✓	✓		✓
		Hospital emergency department	✓	✓		✓
	Medication	Medication	✓			✓
	Genetic testing	Diagnostic test	✓	✓		✓
		Genetic counselor or genetic counsellor visit	✓	✓		✓
	Formal care	Formal care		✓		✓
Direct non-healthcare costs		Aids and modifications	✓			✓
Indirect non-healthcare costs		Reduced work participation				✓
		Absenteeism				✓
		Presenteeism				✓
		Informal care				✓
Government support programs				✓	✓	✓

### Data sources and costs calculation

Direct healthcare costs were calculated by applying unit costs to the healthcare resource utilization reported in the patient survey. The medication unit costs are based on the patient survey, all other unit costs were based on clinical and health-economic expert input on clinical practice of the visits and technical exams applied in Belgium. The reported national tariffs were obtained from the publicly available Nomenclature of Medical Performances database of the National Institute for Health and Disability Insurance (NIHDI) (S2 Table).

For the genetic diagnosis, a genetic counselor visit with a lump-sum cost of €281,70 for standard genetic counselling is taken into account for 75% of patients. For the genetic test, a whole exome sequencing (WES)/Gene panel test was applied, a complex molecular genetic test for the detection of a constitutional disorder. Hospitalization costs were based on the Belgian All Patient Refined Diagnosis Related Groups (APR-DRG) [14]. APR-DRG costs represent the amounts reimbursed by compulsory health insurance per hospitalization, which included daily rates for the hospital stay (including ICU), pharmaceuticals (limited to supporting medications only), and other fees, for which the average cost of €2994,51 at the APR-DRG level 073 ‘procedures on eye and orbita’ was taken into account. For an emergency hospitalization the cost of an ICU day of €2160 as reported in Buryneel et al., 2023 was taken into account [15].

For formal care, the proportion of individuals living with an IRD who receive formal care and number of hours of formal care each person with an IRD receives were collected via the survey. The cost was then calculated by multiplying the average hours of formal care with an average hourly wage of €14 based on the average wage in Belgium as reported by Salaryexplorer in early 2024 [16].

The indirect health care costs consist of reduced labour participation, absenteeism and presenteeism and were based on the survey results. A human capital approach was adopted to estimate productivity losses attributable to IRDs. The cost components were calculated as follows:


$${\text{C}}_{\text{rwp}}=({\text{E}}_{\text{gen}}-{\text{E}}_{\text{IRD}})\times {\text{W}}_{\text{avg}}\times {\text{E}}_{\text{gen}}$$

$${\text{C}}_{\text{abs}}={\text{H}}_{\text{abs}}\times {\text{W}}_{\text{hour}}\times \left(\text{1}-{\text{E}}_{\text{IRD}}\right)\times {\text{E}}_{\text{gen}}$$

$${\text{C}}_{\text{pres}}={\text{H}}_{\text{pres}}\times {\text{W}}_{\text{hour}}\times (\text{1}-{\text{E}}_{\text{IRD}})\times {\text{E}}_{\text{gen}}$$

where all variables and their definitions are summarized in Table 2.

**Table 2:** Definition of variables used in cost estimation.

Symbol	Definition
${\text{C}}_{\text{rwp}}$	Cost of reduced working participation
${\text{C}}_{\text{abs}}$	Absenteeism cost
${\text{C}}_{\text{pres}}$	Presenteeism costs
${\text{E}}_{\text{gen}}$	Employment rate of the general population
${\text{E}}_{\text{IRD}}$	Employment rate of individuals living with an IRD
${\text{H}}_{\text{abs}}$	Reported average number of absenteeism hours
${\text{H}}_{\text{pres}}$	Reported average number of presenteeism hours
${\text{W}}_{\text{avg}}$	Average wage
${\text{W}}_{\text{hour}}$	Average hourly wage

For informal care, the same calculation method as formal care was applied. Estimates of employment rates and average weekly wages by age and gender were accessed through Statbel [17]. A complete list of employment rates and earnings per gender was reported in S3 Table.

Government support programs applicable to IRD patients were included in the patient survey, based on clinical and health-economic experts and patient association input. The government support programs related to tax reduction benefits, as well as other support programs at risk of double counting with other cost items (e.g., direct medical costs, productivity loss and/or aids and modifications) were excluded from the cost analysis (see Table 7). The unit costs per government support program were also tabulated in Table 8. As these payments represent a redistribution of resources in the economy, this cost has not been included in the total financial costs of IRDs.

## Results

### Study population

Table 3 describes the socio-demographic and disease characteristics of the study population of 78 individuals with an IRD. From the initial sample of 82 respondents, 4 were excluded from the analysis as these patients indicated they had received gene or cell therapy to treat their condition. An additional 4 responses from parents of children less than 18 years of age with an IRD were excluded due to the limited sample size. Most respondents were in the 55–64 years of age range (29.5%), with a slight male predominance (53.8%), consistent with international trends. RP was the most prevalent condition, affecting 60.3% of respondents. Genetic testing was conducted for 78.2% of those, with 3.8% of respondents tested in the last year.

**Table 3:** Demographics and characteristics of participants (n = 78) in primary data collection survey.

Item	Percentage
Age	
18-24	0%
25-34	2.6%
35-44	11.5%
45-54	24.4%
55-64	29.5%
65-74	25.6%
75 or older	6.4%
Gender	
Male	53.8%
Female	46.2%
Condition	
Retinitis pigmentosa (RP)	60.3%
Leber congenital amaurosis (LCA)	7.7%
Stargardt disease	7.7%
Cone-rod dystrophy	6.4%
Achromatopsia	5.1%
Usher syndrome	3.8%
Cone dystrophy	3.8%
Best disease	2.6%
Choroideremia	1.3%
X-linked retinoschisis	1.3%
Genetic testing for IRDs	
Yes	78.2%
No	21.8%
Genetic testing received in the lifetime	
Once	75.4%
Twice	17.5%
Three times	5.3%
More than three times	1.8%
Disease Severity	
No vision or field of vision	15.9%
Tunnel vision	63.5%
Large visual field with a central zone of blindness	20.6%
Limitation in daily activities	
Not limited at all	0%
Slightly limited	3.8%
Fairly limited	28.2%
Limited	41%
Very limited	26.9%
Employment status	
Full-time	23.1%
Part-time	16.7%
Retired	30.8%
Not working due to IRD	21.8%
Not working for other reasons	7.7%
Impact on mental health	
No not negatively affected at all	19.5%
Yes a bit negatively affected	37.7%
Yes quite negatively affected	33.8%
Yes very negatively affected	9.1%

The results show 63.5% of all respondents reported experiencing tunnel vision and 15.9% reported no vision or field of vision. All respondents experienced impact of the IRD on their daily activities, which were for 41% of patients considered limited and for 26.9% very limited due to their IRD. Consistently, also the impact on employment was significant, with 21.5% not working due to their IRD. The mental health impact was also reported, with 33.8% reporting a significant negative effect and 9.1% experiencing severe impact.

### Prevalence

Table 4 outlines the internationally reported prevalence rate of each IRD condition, which corresponds to the proportion of people in the population with each IRD listed (e.g., 1 case per 1,000 population). By applying these rates to the general population in Belgium in 2023, the combined total number of patients in Belgium with one of the IRDs under study was estimated at 3,476, with retinitis pigmentosa as the most prevalent condition, accounting for 1,579 cases (45.4%). The data sample collected in the patient survey is representative of the IRD population, as the observed distribution across the different IRD conditions, described in Table 3, is aligned with the literature findings depicted in Table 4. Hence, it can be reliably applied to the cost-of-illness model.

**Table 4:** Internationally reported prevalence rate and estimated number of cases in Belgium by condition.

Condition	Prevalence	%	Estimated number of cases in Belgium	Reference	Referred countries and description
Retinitis pigmentosa (RP)	0.0154%	45.4%	1,579	Bertelsen et al (2014) [18]	Prevalence of RP in **Denmark** based on Danish Retinitis Pigmentosa Registry
Stargardt disease	0.0045%	13.2%	459	Runhart et al (2022) [19]	800 patients with STGD1 in **the Netherlands** registered in the Dutch IRD Registry
Usher syndrome	0.0039%	11.4%	396	Bertelsen et al (2014)	Prevalence of usher syndrome in **Denmark** based on Danish Retinitis Pigmentosa Registry
Congenital stationary night blindness (CSNB)	0.0030%	8.9%	310	prevalence of CSNB type 2 in Hove et al (2016) and relative ratio of CSNB type 1 and 2 in Bijveld et al (2013) [20]	39 patients in **the Netherlands** referred by specialists to a Dutch institution for the care of people who are visually impaired or blind.
Leber congenital amaurosis (LCA)	0.0024%	7.0%	243	Bertelsen et al (2014)	Prevalence of LCA in **Denmark** based on Danish Retinitis Pigmentosa Registry
Cone dystrophy	0.0018%	5.4%	187	Relative prevalence of cone dystrophy and stargardt disease observed in Bocquet et al (2013) [21], multiplied by the observed prevalence rate for Stargardt disease in Runhart et al (2022)	Patients from **France** recruited from a specialized outpatient clinic over a 21-year period; 800 patients with STGD1 in **the Netherlands** registered in the Dutch IRD Registry
Cone-rod dystrophy	0.0012%	3.6%	124	Bertelsen et al (2014)	Prevalence of Cone-rod dystrophy in **Denmark** based on Danish Retinitis Pigmentosa Registry
Best disease	0.0008%	2.4%	85	Relative prevalence of Best disease and Stargardt disease observed in eyeGene, multiplied by the observed prevalence rate for Stargardt disease in Runhart et al (2022).	Publicly available registry data in **Canada** for people with IRDs; 800 patients with STGD1 in **the Netherlands** registered in the Dutch IRD Registry
Choroideremia	0.0004%	1.3%	46	Bertelsen et al (2014).	Prevalence of Choroideremia in **Denmark** based on Danish Retinitis Pigmentosa Registry
X-linked retinoschisis	0.0006% (males only)	1.1%	37	Relative prevalence of XLRS and Stargardt disease observed in eyeGene, multiplied by the observed prevalence rate for Stargardt disease in Runhart et al (2022).	Publicly available registry data in **Canada** for people with IRDs; 800 patients with STGD1 in **the Netherlands** registered in the Dutch IRD Registry
Achromatopsia	0.0001%	0.3%	11	Relative prevalence of achromatopsia and Stargardt disease observed in eyeGene. This relative rate is then multiplied by the observed prevalence rate for Stargardt disease in Runhart et al (2021).	Publicly available registry data in **Canada** for people with IRDs; 800 patients with STGD1 in **the Netherlands** registered in the Dutch IRD Registry
**Total**	**0.03%**	**100%**	**3,476**		

### Healthcare resource utilization

The healthcare resource utilization that patients with IRD reported in Belgium is depicted in Table 5. It highlights regular clinical management by general practitioner, ophthalmologist and retina specialist, with on average 1.6, 1.0 and 3.4 visits per year, as well as psychological and physiotherapy follow up with 2.7 and 5.5 visits per year. Pharmaceuticals and complementary treatment such as eye drops and vitamins were only used by 27% of respondents, who reported an average out-of-pocket expenditure of €69.8 per year. As for genetic testing, 4% of respondents received a genetic test during the last year and 15% had a genetic counselor visit.

**Table 5:** Healthcare resource utilization and costs in euro.

Direct Healthcare cost component	Utilization reported in survey per year, n, (range)	Patient unit cost	Public healthcare payer unit cost (INAMI)^14^	Total unit cost (INAMI+patient)
General practitioner visit	1.64 (0-5)	1.5	28.5	30.0
Retina specialist visit (consultation + medical exams)	0.97 (0-6)	3.0	137.6	140.6
Ophthalmologist visit (consultation + medical exams)	3.4 (0-60)	11.7	155.2	166.9
Psychologist or psychiatrist visit	2.68 (0-20)	3.0	53.8	56.8
Physiotherapist or kinesist visit	5.52 (0-52)	2.5	26.1	28.6
Rehabilitation center or center of expertise	0.31 (0-3)	2.2	406.2	408.4
Low vision department	0.86 (0-4)	2.2	138.1	140.3
Counselor professional organization	3.79 (0-25)	7.8	340.9	348.6
Hospitalisation	0.15 (0-2)	0.0	2,994.5	2,994.5
Hospital Emergency Department	0.1 (0-2)	0.0	2,160.0	2,160.0
Medication	0.27	69.8	0.0	69.8
Diagnostic test	0.04	0.0	1,644.7	1,644.7
Genetic counselor or genetic counselor visit	0.15 (0-1)	3.0	238.3	241.3

The majority of respondents received rehabilitation in the expert center, low vision department and/or via a professional counselor of the patient association, with 0.3, 0.9 and 3.8 visits per year. Hospitalization was reported 0.15 times per year in general hospital departments, and 0.1 time per year in intensive care unit.

### Direct Healthcare costs

Total direct healthcare costs associated with IRDs in Belgium in 2023 were estimated to be €15.2M. Among these, rehabilitation represented the largest expenditure category, amounting to €5.4M. This category included a range of services aimed at supporting patients in adapting to loss of vision in daily life, including help by counselors from professional organizations who provided guidance and information support, as well as support from rehabilitation centers and low-vision departments. Specialty care, the second largest component, accounted for €3.5M. This included regular clinical management by retina specialists and ophthalmologists, psychological support and follow-up physiotherapy sessions. As shown in Table 6, ophthalmologist visits accounted for the largest share of this cost category. Formal care costs of €3.3M were based on the proportion of survey respondents who indicated requiring formal care (19.2%) multiplied by the weekly average hours of care of 6.8 hours among recipients of formal care and an assumed average carer wage of €14.00 [16].

**Table 6:** Total direct healthcare costs of IRDs in Belgium in 2023 by cost type (€ million).

Cost type	Sub-type	Total cost	Total cost by cost type	Total cost borne by patients	Total cost borne by public health payer	Total cost borne by regional government
**Primary care**	General Practitioner	€0.17	€0.2	€0.01	€0.16	/
**Specialty care**	Retina specialist visit	€0.47	€3.5	€0.22	€3.30	/
	Ophthalmologist visit	€1.97				
	Psychologist or psychiatric visit	€0.53				
	Physiotherapist or kinesist visit	€0.55				
**Rehabilitation**	Rehabilitation center or center of expertise	€0.44	€5.4	/	/	€5.4
	Low vision department	€0.42				
	Counselor professional organization	€4.59				
**Hospitalization**	Hospitalization	€1.54	€2.3	/	€2.29	/
	Hospital emergency department	€0.75				
**Medication**	Medication	€0.07	€0.1	€0.07	/	/
**Genetic testing**	Diagnostic test	€0.22	€0.35	/	0.35	/
	Genetic counselor or genetic counsellor visit	€0.13				
**Formal care**	Formal care	€3.31	€3.3	/	€3.3	/
**Total**			**€15.2**	**€0.3**	**€9.4**	**€5.4**

Note:/ indicates that this cost category is not applicable/included to the specified payer.

Funding responsibilities varied across cost categories. Rehabilitation services, at €5.4M, were mainly funded by the regional government, while formal care costs (€3.3M) were entirely covered by the public health payer. Medication costs, amounting to €0.1M, were borne solely by patients. The remaining costs, including specialty care and other healthcare services, were co-funded by patients and the public healthcare system. A detailed cost breakdown by category was presented in Table 6.

### Direct non-healthcare costs

Direct non-healthcare costs correspond to the reported out-of-pocket expenses in the survey to aids and modifications, including guide dogs, home modifications, magnifying glasses, global positioning systems, electronic mobility devices, etc., used to assist persons living with an IRD. The reported expenses amounted to €5.2M in 2023, as detailed by item in Table 7. These expenses are covered by patients in their entirety.

**Table 7:** Total direct non-healthcare costs of IRDs in Belgium in 2023 by aids and modifications items.

Aids and modifications	Total cost (€ million, 2023)
Computer tools	€0.2
Reading aids	€0.2
Tools for activities of daily life	€0.4
Optical aids	€0.6
Custom lighting	€0.1
Ergonomic aids	€0.3
Guide dog	€1.5
Tactical or signaling stick	€0.5
Navigation apps/ electronic mobility aids	€0.1
Home modifications	€1.4
**Total**	€5.2

### Indirect non-healthcare costs

**Fig 1:**
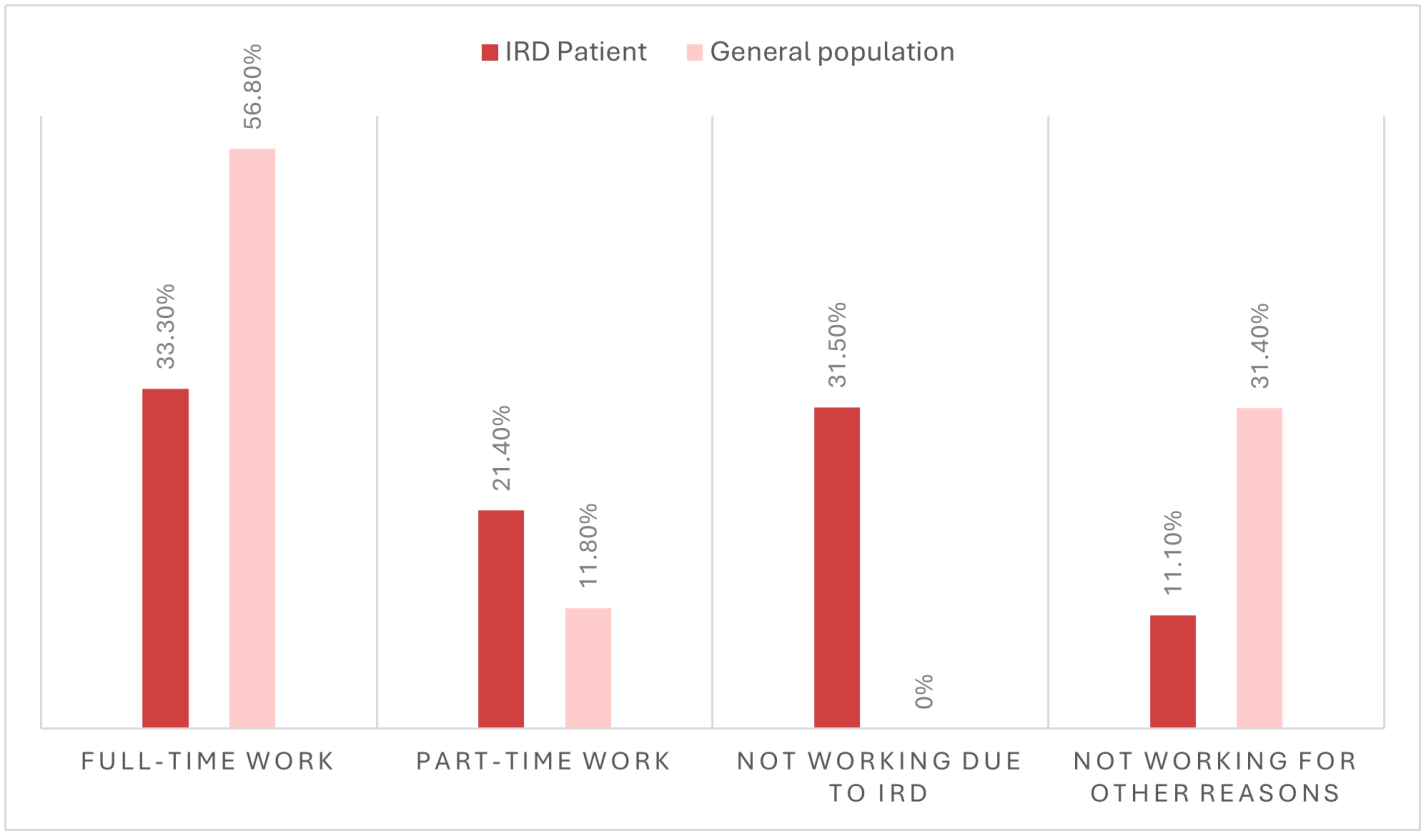
Distribution of Belgian survey respondents by employment status.

Indirect non-healthcare costs consist of reduced working participation, absenteeism, presenteeism and informal care. Patient survey respondents within the working-age experience a high impact on their employment status as depicted in Fig 1, which corresponds to a reduced employment of 28% compared to the general population aged 20–64, which translates into an annual cost of € 32.6M. For absenteeism, an on average reduced presence of 16 days per year and for presenteeism a percentage of 45.5% were reported in the survey, corresponding to € 6.8M and € 38.3M respectively. Informal care costs of € 31.3M were based on the proportion of survey respondents who indicated requiring informal care (76%) multiplied by the weekly average hours of care of 16 hours among recipients of formal care and an assumed average carer wage of €14.00 [16]. Thereby, the total indirect non-health care cost sums up to € 109.0M per year.

### Government support programs

Government support programs were often utilized by the survey respondents, with 42% applying for a card-free accompanist and requesting a personal aid program at the Flemish Agency for Persons with a Disability (VAPH), 29% submitted an application for an invalidity benefit and 25% for an integration allowance. The majority of respondents used the travel allowances such as free bus services, national reduction programs for train tickets and parking cards, as well as multiple social tariffs and/or tax reductions (e.g., reduced TV license fee). To avoid double counting, a conservative approach was used, with only the government support program costs unrelated to aids and modifications, productivity and/or direct medical costs were accounted for (Table 8). When applying the percentage of respondents to the unit cost per program this corresponded to an overall government support program cost of €4.9M annually, of which €3.0M at regional and €1.9M at federal level.

**Table 8:** Government support programs.

	Number of respondents (total = 77)	Proportion of responses	Unit cost per program	Budget owner
**Government support programmes**				
Card free accompanist	32	42%	–	Federal
Requesting personal aid (essential material assistance) from the Flemish Agency for Persons with a Disability (VAPH)	32	42%	–	Regional
Invalidity benefit	22	29%	–	Federal
Integration allowance (IT)	19	25%	–	Federal
Personal budget (PVB)	8	10%	–	Regional
Visual rehabilitation sessions in the center for visual rehabilitation & low vision	8	10%	–	Regional
Income replacement allowance (IVT)	6	8%	–	Federal
Informal care premium (Flanders & Wallonia)	6	8%	600 [22]	Regional
Repayment of labor item adjustments	6	8%	110 [23]	Regional
Third party assistance allowance	5	6%	7,976 [24]	Federal
Flemish support premium (VOP)	4	5%	9,384 [25]	Regional
Allowance for assistance to the elderly (THAB)	3	4%	5,784 [26]	Regional
Basic support budget (PDO)	2	3%	3,600 [27]	Regional
Care budget for those in serious need of care	2	3%	1,620 [28]	Federal
Direct Accessible Help (RTH)	2	3%	–	Regional
Personal assistance budget (PAB)	1	1%	–	Regional
**Tax exemptions and benefits**				
Travel allowances: Free bus	54	70%	–	Federal
Travel allowances: National railroads reduction card	43	56%	–	Federal
Travel allowances: Parking card	42	55%	–	Federal
Property tax reduction	33	43%	–	Regional
Social tariff for gas and electricity	30	39%	–	Federal
Exemption from road tax	24	31%	–	Federal
Exemption from the levy on water pollution	20	26%	–	Regional
Reduction of VAT maintenance and repair	16	21%	–	Federal
Social tariff for fixed telephony or mobile phone	14	18%	–	Federal
Exemption from registration tax for cars	11	14%	–	Federal
Social tariff for cable TV subscription	10	13%	–	Federal
Exemption from car traffic tax	6	8%	–	Federal

### Total costs

**Fig 2:**
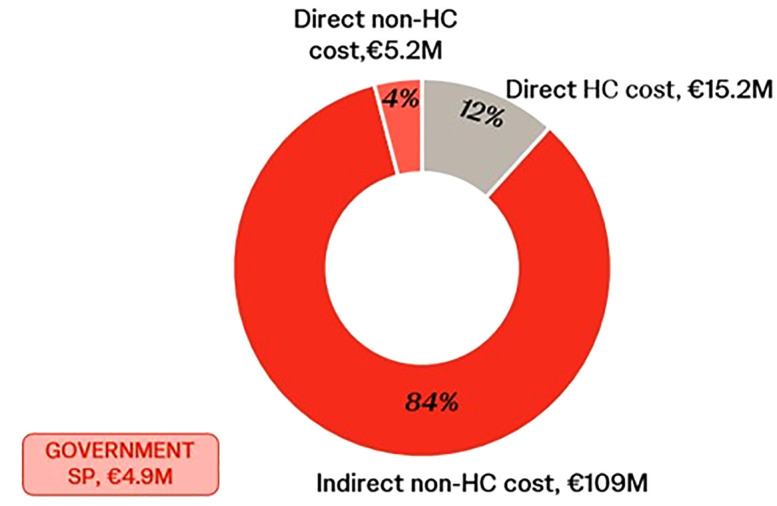
Total cost of IRDs in Belgium (million €).

Total costs attributable to IRDs in Belgium were estimated to be €129.4M in 2023, with an annual cost of €37,228 per patient. Among these costs, indirect non-healthcare costs accounted for the largest share at 84%, followed by direct healthcare cost at 12%, and direct non-healthcare cost at 4% (Fig 2). Of the 11 IRDs within scope, retinitis pigmentosa incurred the greatest proportion of total costs (€58.8M in 2023), followed by Stargardt disease (€16.9M), Usher syndrome (€ 14.5M), and congenital stationary night blindness (€11.8 M). With its relatively high prevalence within the IRD group of rare eye diseases, it is important to note that the results are particularly relevant for the retinitis pigmentosa subgroup, which comprised the majority of survey respondents, underscoring the significant impact of this condition on overall costs. The government support programs related to tax reduction benefits, as well as other support programs were excluded from the total cost calculation. Nevertheless, these programs accounted for €4.9M.

## Discussion

IRDs are a group of genetic disorders which can lead to visual impairment, progressive vision loss and blindness, imposing a substantial humanistic and economic burden on affected individuals and the healthcare system. The study findings reveal that 68% of participants report experiencing limitations in their activities of daily living due to the disease and 81% experience negative mental impact. The mental health status critically highlights the significant and ongoing negative impacts experienced by persons living with an IRD. This statistic is particularly concerning in light of a study indicating that, as of November 2023, 20% of general Belgian population suffers from anxiety disorders, while 18% experience depressive disorders [29]. This stark contrast highlights the unique psychological burden faced by individuals living with chronic, progressive vision loss. Individuals with IRDs are more likely to experience heightened mental health challenges, likely due to the uncertainty surrounding disease progression, often with incrementally increasing functional impairments, and increased social isolation. Individuals with IRDs experience a significant loss of wellbeing and productivity. In line with the reported 45.5% of participants with IRDs being employed in the UK [9], 39.8% of participants with IRDs report employment in Belgium. Consequently, within the eligible working population (aged 20–64), employment was found to be reduced by 28% compared to the general population in Belgium aged 20–64 [30].

The global estimated prevalence of IRDs is estimated to be 1 in 3,000 individuals [12]. In our study, we estimated the prevalence of 11 most prevalent IRDs in Belgium to be 0.03% of the adult population, or 3,476 cases in 2023. The prevalence estimates are based on studies derived from registries in which patients’ diagnosis was confirmed by genetic testing reflecting disease presentation and diagnosis in clinical practice. Despite significant progress in genome sequencing, 20–50% of the genetic basis of IRDs remains unknown despite being analyzed by whole-exome sequencing. This will further improve with the advent of whole genome sequencing, although much work is yet to be done to genetically account for all IRD patients. As current genetic testing rates are suboptimal, the estimated prevalence is likely rather conservative and not expressing the full burden of IRDs in Belgium.

This study aimed to estimate the economic burden on patients, their families, the healthcare system, and government spending in one year. Using a cost‑of‑illness approach based on patient survey data, annual costs in Belgium were estimated at €37,228 per patient, though this is likely to be undervalued/underestimated due to omitted indirect costs, disparities in healthcare access, and possible underreporting of related expenses. The highest cost attributed to IRDs are indirect non-healthcare costs, primarily driven by informal care and reduced working participation. Additionally, healthcare costs are relatively low in the current healthcare landscape considering the lack of effective treatment for IRDs.

There is currently a lack of similar studies that estimate the economic impact of IRDs in Belgium. The here reported healthcare costs of €11.8 million and non-healthcare costs of €124.2 million, are aligned with previously conducted studies in the UK [9] and worldwide [31]. All studies consistently found non-healthcare-related (societal) costs to be the main cost driver (>85%). In terms of absolute costs, a wider range has reported, due to differences in healthcare systems in terms of healthcare expenditure, allocation of costs and the organization and accessibility of healthcare services across countries as well as variation in the specific costs assessed [9,31].

IRDs are significantly underfunded [9] – and increasing awareness of them as a group of conditions would increase government and research bodies’ commitment to funding research and help alleviate the societal costs they impose. Combined with the economic burden, patients with IRDs experience a significant mental burden that could be minimized through expanded governmental support programs offering aid to patients and their families.Given that the societal impact and burden of IRDs in Belgium are likely underestimated, current funding remains disproportionately low compared to their actual economic burden. The needs for testing and care services remain suboptimal, with 78% of survey participants reporting having received genetic testing, highlighting the need for greater investment in these services to help further reduce the societal cost of IRDs. Such investment should also consider reimbursement to individuals and families who bear the greatest cost burden, due to limited treatment availability, and who currently may not benefit sufficiently from health system compensation compared with patients suffering from other conditions.

In summary, the burden of IRDs is substantial, reflecting both their prevalence and their wide-ranging impact on patients’ lives. Our cost‑of‑illness analysis estimated an average annual cost of €37,228 per patient, alongside substantial decline in mental and physical health. This multifaceted burden emphasizes the urgent need for comprehensive support systems to address both the clinical, social and economic challenges faced by individuals with IRDs and their families.

Our study has several limitations, including: (1) the limited availability of estimates of population prevalence of IRDs, and a lack of data on the prevalence in Belgium; (2) the discrepancy in definition of different IRDs in the literature; (3) the retrospective nature of data collection through patient surveys which may have incurred recall bias; (4) the survey data was collected through non-probabilistic convenience sampling data, which may incur sampling bias towards patients who have access to the internet and smart phones or computers. With this taken into consideration, the survey results may not generalize perfectly to the broader IRD community of Belgium.

One aspect which deserves further study in the future is whether significant differences in impact on activities of daily living, mental health and loss of income exist between the different types IRDs. Differentiating between progressive and stationary IRDs as well as between conditions leading to more central or peripheral visual loss may inform about disease specific differences in societal impact of IRDs.

## Conclusions

In conclusion, this study highlights the significant total annual cost of IRDs to Belgian society, estimated to be €129.4M with an annual cost of €37,228 per patient. This significant societal cost is at least in part driven by the lack of research and development and subsequent lack of awareness and paucity of effective therapies available for these rare conditions. These costs could be reduced through improvements in research funding, development of support programs to provide additional aids and programs in mental health to patients and expediting development of and patient access to innovative medicines. Changes at the regulatory level for innovative medicines for IRDs are long overdue as is the development of appropriate approaches to health technology assessment. This is especially true when considering the lower rates of engagement of the IRD community with the health system and the greater proportion of costs borne by individuals, their families and broader society.

## Supporting information

S1 Table Patient survey. (PDF)

S1 Fig Demographics and characteristics of survey participants (n = 78). (PDF)

S2 Table Healthcare resource items and unit costs. (PDF)

S3 Table Employment rates and earnings. (PDF)

## References

[1] Duncan JL, Pierce EA, Laster AM, Daiger SP, Birch DG, Ash JD, et al. Inherited Retinal Degenerations: Current Landscape and Knowledge Gaps. Transl Vis Sci Technol. 2018;7(4):6. doi: 10.1167/tvst.7.4.6 30034950 PMC6052953

[2] Hafler BP. Clinical progress in inherited retinal degenerations: gene therapy clinical trials and advances in genetic sequencing. Retina. 2017;37(3):417–23. doi: 10.1097/IAE.0000000000001341 27753762 PMC5465814

[3] Heath Jeffery RC, Mukhtar SA, McAllister IL, Morgan WH, Mackey DA, Chen FK. Inherited retinal diseases are the most common cause of blindness in the working-age population in Australia. Ophthalmic Genet. 2021;42(4):431–9. doi: 10.1080/13816810.2021.1913610 33939573 PMC8315212

[4] Ben-Yosef T. Inherited Retinal Diseases. Int J Mol Sci. 2022;23(21):13467. doi: 10.3390/ijms232113467 36362249 PMC9654499

[5] Stephenson KAJ, Zhu J, Dockery A, Whelan L, Burke T, Turner J, et al. Clinical and Genetic Re-Evaluation of Inherited Retinal Degeneration Pedigrees following Initial Negative Findings on Panel-Based Next Generation Sequencing. Int J Mol Sci. 2022;23(2):995. doi: 10.3390/ijms23020995 35055178 PMC8780304

[6] Sergouniotis PI. Inherited Retinal Disorders: Using Evidence as a Driver for Implementation. Ophthalmologica. 2019;242(4):187–94. doi: 10.1159/000500574 31280272

[7] Maguire AM, Bennett J, Aleman EM, Leroy BP, Aleman TS. Clinical Perspective: Treating RPE65-Associated Retinal Dystrophy. Mol Ther. 2021;29(2):442–63. doi: 10.1016/j.ymthe.2020.11.029 33278565 PMC7854308

[8] Chivers M, Li N, Pan F, Wieffer H, Slowik R, Leartsakulpanitch J. The Burden of X-Linked Retinitis Pigmentosa on Patients and Society: A Narrative Literature Review. Clinicoecon Outcomes Res. 2021;13:565–72. doi: 10.2147/CEOR.S297287 34188501 PMC8236258

[9] The socioeconomic impact of inherited retinal dystrophies (IRDs) in the United Kingdom. Retina International. 2019. https://www.retina-international.org/wp-content/uploads/2019/11/cost-of-illness-report-uk.pdf

[10] The socioeconomic impact of inherited retinal dystrophies (IRDs) in the Republic of Ireland. 2019. https://retina-international.org/wp-content/uploads/2024/06/The-socioeconomic-impact-of-inherited-retinal-dystrophies-IRDs-in-the-Republic-of-Ireland.pdf

[11] Murro V, Banfi S, Testa F, Iarossi G, Falsini B, Sodi A, et al. A multidisciplinary approach to inherited retinal dystrophies from diagnosis to initial care: a narrative review with inputs from clinical practice. Orphanet J Rare Dis. 2023;18(1):223. doi: 10.1186/s13023-023-02798-z 37525225 PMC10388566

[12] Sahel J-A, Marazova K, Audo I. Clinical characteristics and current therapies for inherited retinal degenerations. Cold Spring Harb Perspect Med. 2014;5(2):a017111. doi: 10.1101/cshperspect.a017111 25324231 PMC4315917

[13] National Institutes of Health. National Ophthalmic Disease Genotyping and Phenotyping Network. https://eyegene.nih.gov/ 2025 May 21.

[14] Technische cel. Nationale Databank Medische Diagnose/ Zorg & Kost. In: Belgium.be. 2008 https://tct.fgov.be/webetct/etct-web/html/nl/index.jsp

[15] Bruyneel A, Bouckaert N, Maertens de Noordhout C, Detollenaere J, Kohn L, Pirson M, et al. Association of burnout and intention-to-leave the profession with work environment: A nationwide cross-sectional study among Belgian intensive care nurses after two years of pandemic. Int J Nurs Stud. 2023;137:104385. doi: 10.1016/j.ijnurstu.2022.104385 36423423 PMC9640385

[16] Salaryexplorere. Care worker average salary in Belgium. https://www.salaryexplorer.com/average-salary-wage-comparison-belgium-care-worker-c21j10031?expand_article=1. 2023. 2025 May 21.

[17] Belgian Federal Government (Statbel). Statbel. Belgium.be. https://statbel.fgov.be/en. 2017. 2025 May 21.

[18] Bertelsen M, Jensen H, Bregnhøj JF, Rosenberg T. Prevalence of generalized retinal dystrophy in Denmark. Ophthalmic Epidemiol. 2014;21(4):217–23. doi: 10.3109/09286586.2014.929710 24963760

[19] Runhart EH, Dhooge P, Meester-Smoor M, Pas J, Pott JWR, van Leeuwen R, et al. Stargardt disease: monitoring incidence and diagnostic trends in the Netherlands using a nationwide disease registry. Acta Ophthalmol. 2022;100(4):395–402. doi: 10.1111/aos.14996 34431609 PMC9291619

[20] Bijveld MMC, Florijn RJ, Bergen AAB, van den Born LI, Kamermans M, Prick L, et al. Genotype and phenotype of 101 dutch patients with congenital stationary night blindness. Ophthalmology. 2013;120(10):2072–81. doi: 10.1016/j.ophtha.2013.03.002 23714322

[21] Bocquet B, Lacroux A, Surget M-O, Baudoin C, Marquette V, Manes G, et al. Relative frequencies of inherited retinal dystrophies and optic neuropathies in Southern France: assessment of 21-year data management. Ophthalmic Epidemiol. 2013;20(1):13–25. doi: 10.3109/09286586.2012.737890 23350551

[22] Van Rossom S. Wanneer heb je recht op een mantelzorgpremie?. Gezondheid.be. https://www.gezondheid.be/artikel/patientenzorg/wanneer-heb-je-recht-op-een-mantelzorgpremie-19999. 2023. 2025 May 21.

[23] VDAB. Tussenkomst werkplekaanpassing. VDAB. https://werkgevers.vdab.be/wegwijs/gezondheidsprobleem/werkplekaanpassing 2025 May 21.

[24] Rijksinstituut voor ziekte- en invaliditeitsverzekering. Hulp van derden. RIZIV. https://www.riziv.fgov.be/nl/thema-s/arbeidsongeschiktheid/werknemers-en-werklozen/hulp-van-derden 2025 May 21.

[25] Vlaanderen. Vlaamse ondersteuningspremie - Bedrag en duur. Vlaanderen.be. https://www.vlaanderen.be/economie-en-ondernemen/personeel/vlaamse-ondersteuningspremie-vop/vlaamse-ondersteuningspremie-bedrag-en-duur 2025 May 21.

[26] Vlaanderen. Zorgbudget voor ouderen met een zorgnood. Vlaanderen.be. https://www.vlaamsesocialebescherming.be/zorgbudget-voor-ouderen-met-een-zorgnood 2025 May 21.

[27] Vlaanderen. Zorgbudget voor mensen met een handicap. Vlaanderen.be. https://www.departementzorg.be/nl/zorgbudget-voor-mensen-met-een-handicap 2025 May 21.

[28] MyIriscare. Questions fréquentes. myiriscare.brussels. https://www.myiriscare.brussels/fr/faq/#quel-montant-puis-je-recevoir 2025. 2025 May 21.

[29] Sciensano. Mental health: Anxiety and depression, health status report. 2020. https://www.healthybelgium.be/en/health-status/mental-health/anxiety-and-depression

[30] Belgian Federal Government (Statbel). Employment and unemployment. Belgium.be. https://statbel.fgov.be/en/themes/work-training/labour-market/employment-and-unemployment 2024. 2025 May 21.

[31] Ng QX, Ong C, Yaow CYL, Chan HW, Thumboo J, Wang Y, et al. Cost-of-illness studies of inherited retinal diseases: a systematic review. Orphanet J Rare Dis. 2024;19(1):93. doi: 10.1186/s13023-024-03099-9 38424595 PMC10905859

