# Supplementary material for: The socioeconomic impact of inherited retinal dystrophies (IRDs) in Belgium: A cost-of-illness study
Source: PLoS One. 2026 Jan 27;21(1):e0339332. doi: 10.1371/journal.pone.0339332 (PMC12843553; doi:10.1371/journal.pone.0339332)
Supplement: S1 Table — (PDF) [file pone.0339332.s001.pdf]

S1 Table. Patient survey. SD, standard deviation.

**Q1: Would you like to complete this questionnaire online or would you rather be called so that you answer the questions over the phone? (n=82)**	
I want to fill it out online	61
I want to be called, please enter your phone number here	21
From the initial sample of 82 respondents in Q1, in the following analysis 4 were excluded as these patients indicated they had received gene or cell therapy to treat their condition.	
**Q2: For whom are you completing this questionnaire? 1 answer possible, 4 options (n=78)**	
Myself as a person with a hereditary retinal disorder	78
As a caregiver of someone with a hereditary retinal disorder	0
As a parent or guardian of a child up to 18 years old with a hereditary retinal disorder	0
None of the above [Screen out]	0
**Q3: What inherited retinal disease have you been diagnosed with? 1 answer possible, 13 options (n=78)**	
Retinitis pigmentosa (RP)	47
Leber congenital amaurosis (LCA) [Skip next question]	6
Usher syndrome [Skip next question]	3
Strandt's disease [Skip next question]	6
Choroideremia [Skip next question]	1
Linked retinoschisis [Skip next question]	1
Achromatopsia [Skip next question]	4
Cone Systrophia [Skip Next Question]	3
Cone rod dystrophy [Skip next question]	5
Best disease [Skip next question]	2
Congenital stationary night blindness - [Skip next question]	0
Otherwise, namely... [Open] [Screen out]	0
I don't know [Screen out]	0
**Q4: What form of retinitis pigmentosa have you been diagnosed with? 1 answer possible, 4 options (n=47)**	
Autosomal dominant RP	14
Autosomal recessive RP	16
X paired RP	2
I don't know	15

**Q5: What was your biological sex at birth? 1 answer possible, 3 options (n=78)**	
Woman	36
Man	42
I'd rather not say	0
**Q6: What is your year of birth? Type in your year of birth here. (n=78)**	
2005-1995	1
1994-1985	7
1984-1975	6
1974-1965	25
1964-1955	23
1954-1945	15
1944-Earlier	1
**Q7: What country do you currently live in? 1 answer possible, 2 options (n=77)**	
Belgium	77
Otherwise namely [Open] [Screen out]	0
**Q8: Which region do you currently live in? 1 answer possible, 3 options (n=76)**	
Flanders	51
Wallonia	15
Brussels	10
**Q9: How is your field of vision? 1 answer possible, 4 options (n=78)**	
I have no vision or field of vision	10
I have tunnel vision	40
I have a large field of vision but with a central zone of Blindness	13
Other, namely [Open]	15
**Q10: How is your best corrected visual acuity? With glasses or lenses and with your best eye. 1 answer possible, 5 options (n=78)**	
I have no vision or field of vision	15
I couldn't read the letters on the reading card with letters at my last ophthalmologist visit (OR visual acuity 1/10)	23
I could read some letters on the card, but with difficulty (OR visual acuity between 2/10-5/10)	15
I could read the letters on the card and may have made a few mistakes (OR visual acuity > 5/10)	23
I don't know	2

**Q11: To what extent do you feel limited in your daily activities because of your vision. 1 answer possible, 5 options (n=78)**	
Not limited at all	0
A bit limited	3
Fairly limited	22
Limited	32
Very limited	21
**Q12: Can you describe in 1 or 2 sentences how you feel emotionally about living with an eye condition?** *Open question, type in your answer. [Qualitative inputs]*	
**Q13: Has your state of mind or your mental health been negatively affected by your eye condition in the past 12 months? 1 answer possible, 4 options (n=77)**	
No not negatively affected at all	15
Yes a bit negatively affected	29
Yes quite negatively affected	26
Yes very negatively affected	7
**Q14: Has a doctor or specialist diagnosed hearing loss? 1 answer possible, 2 options (n=78)**	
Yes	21
No [Skip next question]	57
**Q15: Are you currently using a hearing aid or other device because of your hearing loss? 1 answer possible, 4 options (n=21)**	
Yes I use an external hearing aid	6
Yes I use a cochlear implant	0
No I am not currently using a hearing aid	10
Other. Please fill in your answer	5
**Q16: Are you currently working? 1 answer possible, 2 options (n=78)**	
Yes [Skip next question]	31
No	47
**Q17:Why do you not work?** *[Qualitative inputs]*	
**Q18: How many hours do you work on average in a regular week? [Open] hours (n=31)**	
1-20	7
21-40	21
41+	3
**Q19: How many hours have you worked in total in the past 4 weeks? Please enter the number of hours below. Open question. (n=29)**	
1-79	7

80-159	13
160+	9
**Q20: Now think about your work experiences over the past 4 weeks. How many hours have you missed in the past 4 weeks because of your eye condition compared to your regular work week? Only mention the missed hours due to your own health, not anyone else's. Enter the number of hours below. Open question. (n=30)**	
0	24
10-59	4
60+	2
**Q21: To what extent has your condition affected your productivity during the last 7 days while you were working? Think of hours when you were limited in the amount or type of work you could do. Or the hours when you did less than you'd like or days when you couldn't do your job as carefully as usual. If your eye condition affected your work only slightly then choose a low number. Choose a high number if your condition has had a significant impact on your work. Open question. Enter your number between 0 and 10 here, where 0 is no influence at all and 10 is very influential. (n=31)**	
0-2	9
3-6	13
7-10	9
**Q22: Which of the following means of transport do you use to get to work? Multiple answers possible, 6 options (n=28)**	
Car with or without driver	9
Bicycle [Continue to module 3]	4
Public transport such as bus train tram	17
Taxi	3
Ongoing [Continue to Module 3]	9
I work from home [Continue to module 3]	16
**Q23: What are your transport costs to work each week? 1 answer possible, 5 options (n=9)**	
€0 to €40	7
€41 to €80	1
€81 to €120	1
€120 or more	0
I work from home	0
**Q24: Will your travel expenses be reimbursed? 1 answer possible, 5 options (n=9)**	
Yes partly paid by my employer	3
Yes fully paid by my employer	1

Yes partly paid by the government	0
Yes fully paid by the government	2
No	3
**Q25: Are you currently following any education or training? 1 answer possible, 2 options (n=78)**	
Yes	7
No [Continue to module 4]	71
**Q26: Where are you currently following your training? 1 answer possible, 5 options (n=7)**	
Primary education	0
Secondary school	0
Higher education such as college or university	2
Other educational body. Enter the educational authority here.	5
**Q27: Because of your eye condition, do you receive extra support from a supervisor in your learning environment, such as a General Education Supervisor or an itinerant educational supervisor? 1 answer possible, 2 options (n=7)**	
Yes	1
No [Continue to module 4]	6
**Q28: How many hours do you receive extra support from a supervisor on average per week? 1 answer possible, 4 options (n=1)**	
1 to 5 hours	1
5 to 10 hours	0
10 to 20 hours	0
More than 20 hours	0
**Q29: Have you received a diagnosis confirmed by a genetic test for your inherited retinal disease? For example, identification of a disease-causing gene? 1 answer possible, 3 options (n=78)**	
Yes in the last 12 months	3
Yes more than 1 year ago	58
No [Skip next 2 questions]	17
**Q30: How many genetic tests have you had in your life? Open question please fill in the number (n=57)**	
1	43
2	10
3	3
5	1

**Q31: Approximately how much money did you spend on travel to perform your genetic test and receive the results? For example, petrol, bus costs, train costs or taxi costs. If you are not sure then answer not applicable. 1 answer possible, 7 options (n=61)**	
Not applicable	25
€0 to €100	22
€101 to €500	13
+€500	1

**Q32: Have you seen healthcare providers specifically for your eye condition in the past 12 months? For example, doctors or psychologists. 1 answer possible, 3 options (n=78)**	
Yes	48
No [Continue to module 5]	30
Don't know [Continue to module 5]	0

**Q33-34:**	**How many times have you used a service for your eye condition in the last 12 months? (n=48)**	**Approximately how much money did you pay on your last visit in the last 12 months to the health care provider outside of government reimbursements and financial support? (n=48)**
General practitioner	***Mean***: 1,64 (***SD***: 1,82) ***Median:*** 1 [0-5] (n=33)	***Mean***: 14,38 (***SD***: 28,71) ***Median:*** 3,5 [0-120] (n=24)
Retina specialist	***Mean***: 0,97 (***SD***: 1,22) ***Median:*** 1 [0-6] (n=34)	***Mean***: 55,57 (***SD***: 98,49) ***Median:*** 0 [0-365] (n=23)
Ophthalmologist	***Mean***: 3,43 (***SD***: 9,36) ***Median:*** 1 [0-60] (n=40)	***Mean***: 55,22 (***SD***: 47,91) ***Median:*** 50 [0-200] (n=36)
Optometrist or optician	***Mean***: 20,58 (***SD***: 107,54) ***Median:*** 1 [0-600] (n=31)	***Mean***: 190,09 (***SD***: 283,76) ***Median:*** 0 [0-800] (n=23)
Counselor of professional organization such as Brailleliga and Light and Love	***Mean***: 3,79 (***SD***: 5,78) ***Median:*** 1 [0-25] (n=33)	***Mean***: 5,21 (***SD***: 17,16) ***Median:*** 0 [0-70] (n=24)

Psychologist or psychiatrist	***Mean***: 2,68 (***SD***: 5,45)<br>***Median:*** 0 [0-20]<br>(n=28)	***Mean***: 69,21 (***SD***: 249,07)<br>***Median:*** 0 [0-1080]<br>(n=19)
Genetic counselor or genetic counselor	***Mean***: 0,15 (***SD***: 0,37)<br>***Median:*** 0 [0-1]<br>(n=26)	***Mean***: 0,35 (***SD***: 1,00)<br>***Median:*** 0 [0-3]<br>(n=17)
Physiotherapist or kinesist	***Mean***: 5,52 (***SD***: 11,29)<br>***Median:*** 0 [0-52]<br>(n=29)	***Mean***: 83,25 (***SD***: 154,43)<br>***Median:*** 0 [0-500]<br>(n=20)
Hospital Emergency Department	***Mean***: 0,08 (***SD***: 0,39)<br>***Median:*** 0 [0-2]<br>(n=26)	***Mean***: 6,67 (***SD***: 23,76)<br>***Median:*** 0 [0-100]<br>(n=18)
Hospitalised	***Mean***: 0,15 (***SD***: 0,46)<br>***Median:*** 0 [0-2]<br>(n=27)	***Mean***: 5,05 (***SD***: 22,35)<br>***Median:*** 0 [0-100]<br>(n=20)
Low vision department	***Mean***: 0,86 (***SD***: 1,15)<br>***Median:*** 0 [0-4]<br>(n=28)	***Mean***: 7,68 (***SD***: 24,15)<br>***Median:*** 0 [0-100]<br>(n=19)
Rehabilitation centre or centre of expertise	***Mean***: 0,31 (***SD***: 0,74)<br>***Median:*** 0 [0-3]<br>(n=26)	***Mean***: 5,15 (***SD***: 22,34)<br>***Median:*** 0 [0-100]<br>(n=20)
**Q35: How long does it take to travel to your healthcare provider to care about your eye condition? Estimate this time for the healthcare provider you visit most often for your eye condition. 1 answer possible, 5 options (n=46)**		
Less than 15 minutes	2	
Between 15 minutes and 30 minutes	13	
Between 30 minutes and an hour	15	
Between 1 hour and 2 hours	11	
More than 2 hours	5	
**Q36: Do you take time off from work or education to go to your healthcare provider's appointments? 1 answer possible, 6 options (n=48)**		
Yes for all my appointments	10	
Yes for about 3 out of 4 of my appointments	1	
Yes for about half of my appointments	1	
Yes for about 1 in 4 of my appointments	1	

No I do not take time off from work or training to attend my appointments	4
Not applicable I don't work or study	31
**Q37: Do you visit the healthcare provider alone or with a friend, family member or professional carer? 1 answer possible, 4 options (n=48)**	
I go to the healthcare provider alone [Continue to module 5]	12
I go to the healthcare provider with a friend or family member	31
I go to the healthcare provider with a formal caregiver [Continue to module 5]	2
I go to the healthcare provider with a friend or family member and a formal caregiver	3
**Q38: Does your friend or family member take time off from work to go to your healthcare provider's appointments with you? 1 answer possible, 5 options (n=34)**	
Yes for all my appointments	9
Yes for about three-quarters of my appointments	1
Yes for about half of my appointments	3
Yes for about a quarter of my appointments	4
No the person attending the visits with me has no work obligations	17
**Q39: Do you know of therapies for hereditary retinal diseases to improve your vision or slow down your vision loss such as gene or cell therapy? 1 answer possible, 4 options (n=78)**	
No I am not aware of these therapies	40
Yes I am aware of these therapies but I have not been treated	37
Yes I am aware of these therapies and I have received treatment	0
I don't know	1
**Q40: Are you taking medicines, vitamins or dietary supplements as part of your eye condition? 1 answer possible, 2 options (n=77)**	
Yes	22
No [Proceed to Module 6]	55
**Q41: What medications, vitamins or dietary supplements do you use as part of your eye condition? Multiple answers possible, 5 options (n=23)**	

Dexamethasone	0
Diamox	1
Eye drops	14
Vitamins and supplements	10
Other, please enter your answer here	4

Q42: On average, how much money have you spent on medications, vitamins or dietary supplements specific to your eye condition in the last 12 months? Outside of repayment and financial support from the government. Please only include what you pay at your own expense. 1 answer possible, 7 options. (n=22)

€ 0	1
€1 to €50	6
€51 to €100	5
+€100	10

Q43-44-45: This question is about devices and adaptations that you use for your hereditary retinal disorder. Multiple answers possible and 25 tools in the list

.	**Click only on the tools you have used in the last 12 months. (n=77)**	**Please indicate here how much money the tools cost for you personally.**	**Which of the tools have you purchased in the last 12 months? (n=76)**
**COMPUTER TOOLS**			
Modified laptops just for you	23	***Mean***: 561,76 (***SD***: 605,32) ***Median:*** 300 [0-1800] (n=17)	3
Reading aloud software	35	***Mean***: 386,84 (***SD***: 861,63) ***Median:*** 0 [0-3000] (n=19)	2
Screen magnification technology	37	***Mean***: 356,67 (***SD***: 666,24) ***Median:*** 0 [0-2000] (n=15)	3
Large keyboards	20	***Mean***: 60,00 (***SD***: 66,80) ***Median:*** 45 [0-200] (n=18)	4

**READING AIDS**			
Braille display	7	***Mean***: 71,43 (***SD***: 188,98) ***Median:*** 0 [0-500] (n=7)	2
Book alternatives such as audiobooks or Braille books	33	***Mean***: 213,22 (***SD***: 627,56) ***Median:*** 0 [0-3000] (n=23)	7
Books with enlarged font	9	***Mean***: 0,00 (***SD***: 0,00) ***Median:*** 0 [0-0] (n=6)	3
OrCam	4	***Mean***: 1000,00 (***SD***: 2213,59) ***Median:*** 0 [0-5500] (n=6)	2
Webbox	5	***Mean***: 180,00 (***SD***: 211,66) ***Median:*** 140 [0-500] (n=6)	1
**TOOLS ACTIVITIES DAILY LIFE**			
Labels with tactile or large letters	8	***Mean***: 136,25 (***SD***: 166,73) ***Median:*** 75 [10-500] (n=8)	2
Custom clocks or timers	19	***Mean***: 114,71 (***SD***: 149,74) ***Median:*** 50 [0-600] (n=17)	3
Mobile phone adjustments such as applications or apps	39	***Mean***: 148,27 (***SD***: 288,01) ***Median:*** 0 [0-1200] (n=22)	5
Talking calculator	9	***Mean***: 34,38 (***SD***: 39,95) ***Median:*** 25 [0-100] (n=8)	1
Label reader	11	***Mean***: 155,71 (***SD***: 168,31) ***Median:*** 100 [0-500] (n=7)	3
Magnifying mirrors	9	***Mean***: 8,57 (***SD***: 14,92) ***Median:*** 0 [0-35] (n=7)	2

Custom mobile phone	26	***Mean***: 508,86 (***SD***: 442,70) ***Median:*** 450 [0-1500] (n=22)	6
Custom bike like tandem	24	***Mean***: 2430,00 (***SD***: 2471,78) ***Median:*** 1500 [0-8000] (n=19)	2
**OPTICAL AIDS**			
Prescription glasses	43	***Mean***: 554,06 (***SD***: 440,35) ***Median:*** 600 [0-1450] (n=32)	11
Contact lenses	5	***Mean***: 100,00 (***SD***: 141,42) ***Median:*** 0 [0-300] (n=5)	2
Magnifying glasses	23	***Mean***: 187,95 (***SD***: 444,74) ***Median:*** 50 [0-2100] (n=22)	8
Sunglasses with filters such as contrast enhancing	40	***Mean***: 418,48 (***SD***: 566,76) ***Median:*** 300 [0-3000] (n=29)	13
Contrast magnifying filters	5	***Mean***: 117,50 (***SD***: 191,20) ***Median:*** 35 [0-400] (n=4)	1
Magnifiers	30	***Mean***: 80,33 (***SD***: 91,35) ***Median:*** 60 [0-250] (n=15)	10
**CUSTOM LIGHTING**			
High intensity lamps	25	***Mean***: 299,05 (***SD***: 593,45) ***Median:*** 100 [0-2500] (n=19)	8
**ERGONOMIC AIDS**			
Ergonomic adjustments at work, for example raised screens	10	***Mean***: 285,71 (***SD***: 429,84) ***Median:*** 0 [0-1000] (n=7)	1

Ergonomic adjustments at home, for example, raised displays	17	***Mean***: 589,29 (***SD***: 1290,38)<br>***Median:*** 200 [0-5000] (n=14)	2
Other... [Open]	18	***Mean***: 2435,63 (***SD***: 4842,21)<br>***Median:*** 200 [0-17000] (n=16)	2

**Q46: Do you own a guide dog? 1 answer possible, 2 options (n=78)**	
Yes	9
No [Skip the following 3 questions]	69
**Q47: Have you had any training to learn how to work with your guide dog from a trained professional? 1 answer possible, 3 options (n=9)**	
Yes, in the last 12 months	0
Yes, more than 1 year ago	7
No	2
**Q48: Approximately how many days did you spend learning to work with your guide dog from a trained professional? If you have not received training, please answer not applicable. 1 answer possible, 9 options (n=9)**	
Not applicable	2
1 day	0
2 days	0
3 to 5 days	1
5 to 7 days	1
7 to 10 days	1
10 to 14 days	1
14 to 30 days	2
More than 30 days	1
**Q49: Approximately how much money have you spent in the past month caring for your guide dog outside of health insurance or government reimbursement? 1 answer possible, 5 options (n=8)**	
€ 0	1
€1 to €19	0
€20 to €49	0
€50 to €100	1
More than €100	6

**Q50: Do you use a tactile stick or a signalling stick to promote your mobility? 1 answer possible, 2 options (n=78)**		
Yes	60	
No [Skip the following 2 questions]	18	
**Q51: Have you taken training to learn how to use your cane? 1 answer possible, 3 options (n=60)**		
Yes, in the last 12 months	10	
Yes, more than 1 year ago	39	
No [Skip the next question]	11	
**Q52: How many days in total have you spent learning to use your stick with a trained professional? If you have not received training, please answer not applicable. 1 answer possible, 6 options (n=49)**		
		In last 12 months (n=10)
Not applicable	1	0
1 day	8	3
2 days	2	0
3 to 5 days	7	1
6 to 7 days	8	1
More than 7 days	23	5
**Q53: Do you use navigation apps or other electronic mobility aids such as GPS or sensors? 1 answer possible, 2 options (n=78)**		
yes	33	
No [Skip the following 4 questions]	45	
**Q54: What were the estimated costs for you of the navigation apps or other electronic mobility aids? If you're not sure, please answer not applicable. 1 answer possible, 5 options (n=33)**		
		In last 12 months (n=10)
Not applicable	13	3
€ 0	9	2
€1 to €49	5	1
€50 to €99	0	0
€100 to €200	1	1
More than €200	5	3
**Q55: Have you purchased these tools in the last 12 months? 1 answer possible, 2 options (n=33)**		
Yes	9	

No	24	
**Q56: Have you received training from a professional supervisor to learn how to handle your mobility aid? 1 answer possible, 3 options (n=33)**		
Yes, in the last 12 months	5	
Yes, more than a year ago	4	
No [Skip next question]	24	
**Q57: Approximately how much time did you spend with the professional supervisor to learn how to handle your mobility aid (other than the tactile stick)? If you have not received training, please answer not applicable. 1 answer possible, 7 options (n=9)**		
		In last 12 months (n=5)
Not applicable	0	0
Less than 1 day	3	1
1 day	1	1
2 days	2	1
3 to 5 days	0	0
6 to 7 days	0	0
More than 7 days	3	2
**Q58: Do you have specific adjustments to your home for your eye condition? For example, lighting or adjustments in the kitchen. 1 answer possible, 3 options (n=78)**		
Yes, in the last 12 months	12	
Yes, more than 1 year ago	28	
No [Continue to module 7]	38	
**Q59: Estimate the cost of these adjustments. If you're not sure, please answer not applicable. 1 answer possible, 7 options (n=40)**		
		In last 12 months (n=12)
Not applicable	14	4
€ 0	0	1
€1 to €100	1	1
€101 to €500	7	3
€501 to €1000	1	1
€1001 to €5000	8	2
More than €5000	9	4
**Q60: Do you have a formal healthcare provider or a person who is paid to provide care to assist you in your daily activities? Specifically because of your eye condition? 1 answer possible, 2 options (n=78)**		
Yes	15	

No [Continue to module 8]	63
**Q61: How many hours a week do you get help from a formal healthcare provider specifically or partly for your eye condition? 1 answer possible, 6 options (n=15)**	
Less than 5 hours	7
5 to 9 hours	4
10 hours to 19 hours	3
20 hours to 29 hours	1
30 hours to 39 hours	0
40 hours or more	0
**Q62: Do you get help from a caregiver? A caregiver includes any person, such as a family member friend or neighbor who provides regular and ongoing assistance to another person without payment for the care provided. If so, from whom? Indicate your most engaged caregiver if you have more than one caregiver. 1 answer possible, 4 options (n=78)**	
Spouse or partner	43
Relative	13
Friend or other acquaintance	3
I am not getting help from a caregiver [Continue to module 9]	19
**Q63: How many hours a week do you get help from a caregiver? 1 answer possible, 6 options (n=59)**	
Less than 5 hours	18
5 hours to 9 hours	8
10 hours to 19 hours	11
16 hours to 29 hours	8
30 hours to 39 hours	1
40 hours or more	13
**Q64: Is your caregiver employed and, if so, in what capacity? Choose the option as it applies to your most engaged caregiver if you have more than 1 caregiver. 1 answer possible, 8 options (n=52)**	
Full-time work	18
Part-time work	6
Retired	19
My caregiver is a student	2
My caregiver has no work and is not looking for work	6

My caregiver has no work and is looking for work	1
**Q65: Has your employer received VOP (Flemish support premium) in the past 12 months? VOP compensates for any additional costs and lower productivity of the employee's disability. 1 answer possible, 4 options (n=78)**	
I'm Not Working [Skip next questions]	46
Yes, my employer receives a support premium	11
No, my employer did not receive [Skip next question]	13
I don't remember/don't know [Skip next question]	8
**Q66: Indicate the approximate monthly amount of the aid premium. 1 answer possible, 5 options (n=11)**	
From 0 to 500 euros	2
From 501 euros to 1500 euros	0
From 1501 euros to 2500 euros	1
From 2501 euros to 3000 euros	0
I don't know	8
**Q67: Has your employer made any changes to the work environment in the last 12 months due to your disability? If so, can you indicate if you remember the kind of changes they made to the work environment to support you? (n=78)**	
I don't work	46
Yes and the changes include [Open]	12
No my employer has not made changes	19
I don't remember/don't know	1
**Q68: Have you received money through tax breaks and exemptions in the last 12 months? These include a reduction in property tax, reduced rate for cars (purchase or maintenance), exemption from road tax and registration tax, social rate for gas and electricity, social rate for fixed telephony and fixed internet. (n=77)**	
Yes, I have	54
No I didn't receive anything	22
I don't remember/don't know	1
**Q69: Are you currently receiving government support in Belgium specifically for your hereditary retinal disease? If so, which of the following forms of assistance have you used in the last 12 months? Multiple answers possible (n=77)**	

Informal care premium (Flanders & Wallonia)	6
Care budget for those in serious need of care	2
Personal assistance budget (PAB)	1
Invalidity benefit	22
Visual rehabilitation sessions in the center for visual rehabilitation & low vision	8
Personal budget (PVB)	8
Allowance for assistance to the elderly (THAB)	3
Basic support budget (PDO)	2
Income replacement allowance (IVT)	6
Integration allowance (IT)	19
Direct Accessible Help (RTH)	2
VAPH for requesting a personal aid (essential material assistance)	32
Exemption from the levy on water pollution	20
National reduction card	43
Free bus	54
Card free accompanist	32
Parking card	42
Social rate fixed line or mobile phone	14
Reduction of cable TV subscription fee	10
Exemption VAT purchase car	11
Reduction of VAT maintenance and repair	16
Exemption from road tax	24
Exemption from car traffic tax	6
Social tariff for gas and electricity	30
Help to third parties	5
Property tax reduction	33
Flemish support premium (VOP)	4
Repayment of labour item adjustments	6

Reimbursement of special educational resources	0
Other	4
**Q70: Can you make a rough estimate of the revenue you have received through government support in the last 12 months? [Open] (n=29)**	
0	8
€1 to €100	3
€101 to €500	2
€501 to €1000	3
€1001 to €5000	8
More than €5000	5
**Q71: Can you indicate in your own words what personal costs you incur or have incurred as a result of your eye condition? Think of personal costs such as a window that breaks or the vacuum cleaner or costs that you incur if you lose things. Can you make a rough estimate of what costs you have incurred throughout your life?** *[Open] / qualitative inputs*	
**Do you have any questions or comments regarding this research?**	

Footnote on data processing:

- Total number of responders were 82.The responders who received a gene therapy for the treatment of their condition (n=4) were excluded from the analysis (except Q1).
- Therefore the total responders included in the final analysis were 78
    - For the table, when the n equals to 78, no specifications added
    - When there is a difference, the respective cohorts are specified
- The formula for the associated total cost with the use of devices is the cost comes from the proportion of people using the item (Q43) multiplied by Average cost of the item (Q44) multiplied by the number of the items purchased each year (Q45). During data cleaning, there was one mismatch entry for magnifiers which did not allow to exclude responses from Luxturna cohort (n=4).
- For questions that required qualitative inputs, there was no narrative analysis in scope; the inputs were used to contextualise the discussion section only.
