# Supplementary material for: The socioeconomic impact of inherited retinal dystrophies (IRDs) in Belgium: A cost-of-illness study
Source: PLoS One. 2026 Jan 27;21(1):e0339332. doi: 10.1371/journal.pone.0339332 (PMC12843553; doi:10.1371/journal.pone.0339332)

AGE DISTRIBUTION

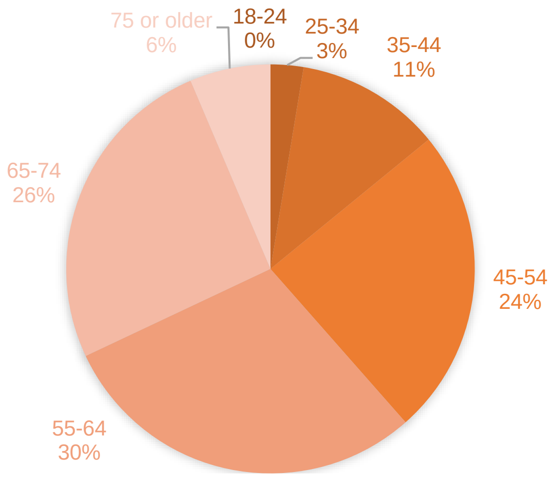

GENDER DISTRIBUTION

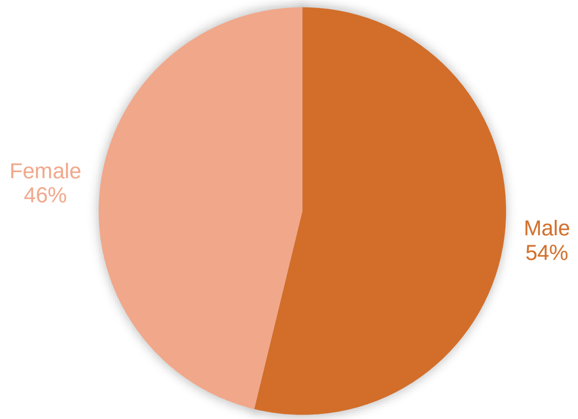

CONDITION DISTRIBUTION

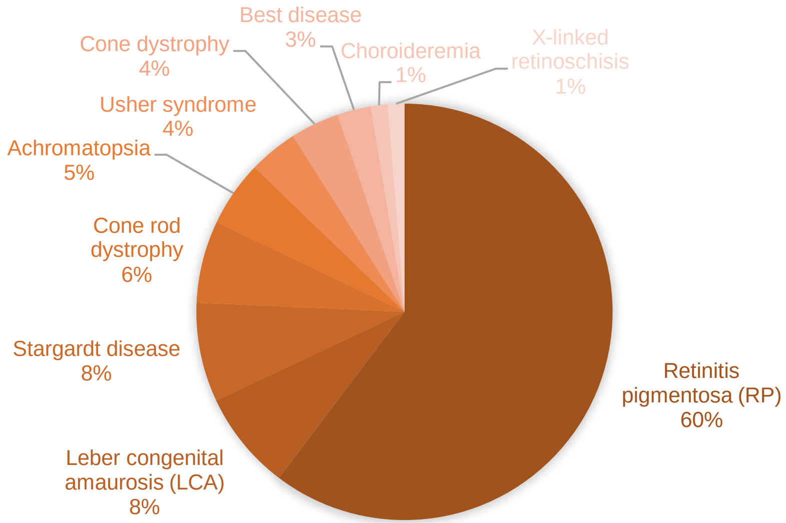

DISEASE SEVERITY DISTRIUTION

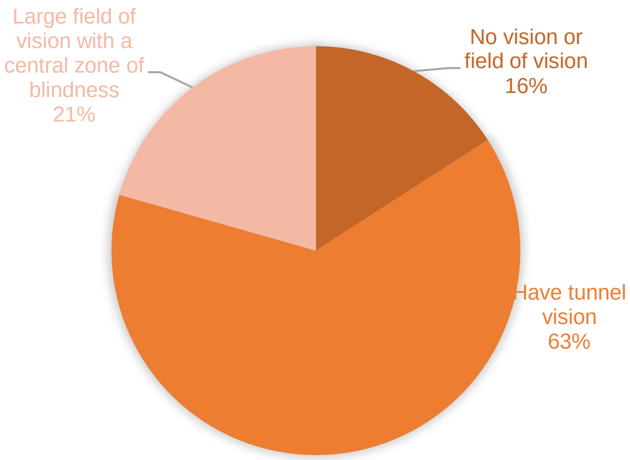

EMPLOYMENT STATUS DISTRIBUTION

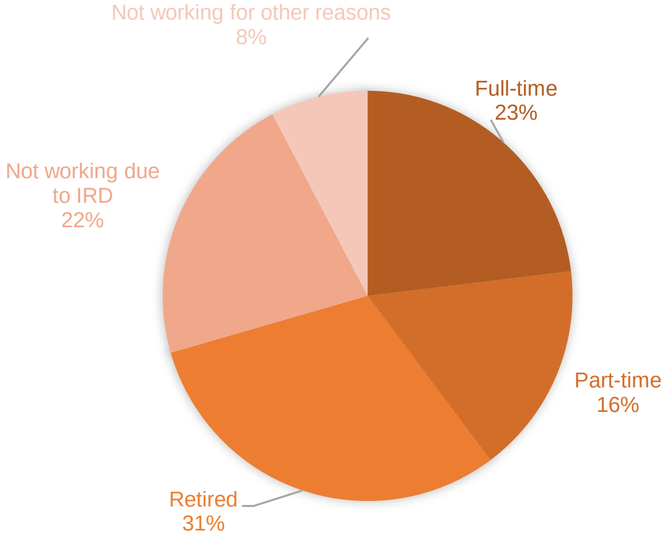

GENETIC TESTING FOR IRD DISTRIBUTION

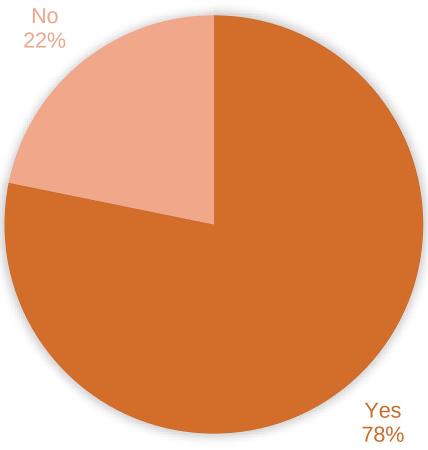

GENETIC TESTING RECEIVED IN LIFETIME DISTRIBUTION

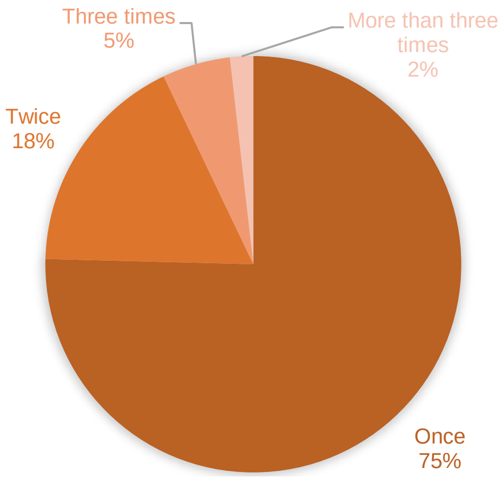

LIMITATION IN DAILY ACTIVITIES DISTRIBUTION

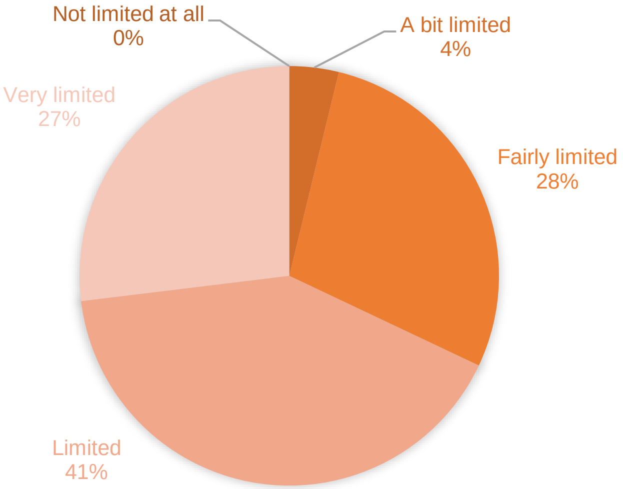

Supplement: S1 Fig — (PDF) [file pone.0339332.s002.pdf]
