# Supplementary material for: The socioeconomic impact of inherited retinal dystrophies (IRDs) in Belgium: A cost-of-illness study
Source: PLoS One. 2026 Jan 27;21(1):e0339332. doi: 10.1371/journal.pone.0339332 (PMC12843553; doi:10.1371/journal.pone.0339332)
Supplement: S2 Table — (PDF) [file pone.0339332.s003.pdf]

S2 Table. Healthcare resource items and unit costs.

Component	Proportion of patients	Patient cost	Public health payer cost	Total cost	Nomenclature	Average number of visits (survey data)
**General practitioner**	**-**	**€ 1.50**	**€ 28.50**	**€ 30**	**101076**	
**Retina specialist**	**-**	**€ 3.00**	**€ 144.50**	**€ 147.50**		**1.0**
└Consultation	-	€ 3.00	€ 27.00	€ 30.00	105755	
└Medical examinations:						
-Electroretinography	33%	€ 0	€ 65.55	€ 65.55	248533	
-OCT	100%	€ 0	€ 40.91	€ 40.91	248356/248360	
-Dyschromatopsias	100%	€ 0	€ 13.11	€ 13.11	248754	
-Quantitative perimetry	100%	€ 0	€ 17.48	€ 17.48	248813	
-Binocular ophthalmoscopy	100%	€ 0	€ 8.74	€ 8.74	248975	
-Binolcular biomicroscopy	100%	€ 0	€ 8.74	€ 8.74	249233	
**Ophthalmologist**	**-**	**€ 18.50**	**€ 129**	**€ 147.50**		**3.4**
└Consultation	-	€ 3.00	€ 27.00	€ 30.00		
∟Medical examinations:	-	€ 8.7	€ 61.9	€ 70.6		
-OCT	50%	€ 0	€ 40.91	€ 40.91	248356/248360	
-Dyschromatopsias	100%	€ 0	€ 13.11	€ 13.11	248754	
-Quantitative perimetry	50%	€ 0	€ 30.59	€ 30.59	249211/249222	
-Binocular ophthalmoscopy	100%	€ 0	€ 8.74	€ 8.74	248975	
-Binolcular biomicroscopy	100%	€ 0	€ 8.74	€ 8.74	249233	
**Counselor professional organization (Braille liga, Light & Love)**	**-**	€ weighted average	**€ weighted average**	**€ weighted average**		**3.8**
∟Via government funding: PVB or RTH	90%	€5.56	€155	€160.56		
∟Via rehabilitation service	10%	€ 2.2	€ 185.85	€ 188.05	251078/251089	
**Psychologist or psychiatrist**		**€ 3**	**€ 53.80**	**€ 56.80**	**102690**	**2.7**

**Genetic counselor or genetic counsellor**		**€ 3**	**€ 27**	**€ 30**	**106470**	**0.2**
∟Forfait standard genetic counselling	75%	€ 0	€ 281.7	€ 281.7	589750	
**Physiotherapist or kinesist**		**€ 2.50**	**€ 26.10**	**€ 28.60**	**567011**	**5.5**
**Hospital Emergency Department**		**-**	**€ 2,160**	**€ 2,994.51**		
**Hospitalization**		**-**	**€ 2,994.51**	**€ 2,994.51**	**APR-DRG 073**	
**Rehabilitation center or center of expertise -Bilan**		**€ 2.2**	**€ 406.15**	**€ 408.35**	**251019/251023**	
**Low vision department**		**€ 2.2**	**€138.14**	**€140.34**	**251056/251067**	
