# Supplementary material for: The socioeconomic impact of inherited retinal dystrophies (IRDs) in Belgium: A cost-of-illness study
Source: PLoS One. 2026 Jan 27;21(1):e0339332. doi: 10.1371/journal.pone.0339332 (PMC12843553; doi:10.1371/journal.pone.0339332)
Supplement: S3 Table — (PDF) [file pone.0339332.s004.pdf]

S3 Table. Employment rates and earnings. Depicted per gender, Belgium 2023.

		Employment rate	*Average weekly earnings (€)*
Male	*15-19*	32.4%	665.18
	20-24	32.4%	743.27
	25-29	90.7%	860.68
	30-34	90.7%	979.79
	35-39	90.7%	1,082.32
	40-44	90.7%	1,171.92
	45-49	90.7%	1,221.92
	50-54	71.9%	1,254.79
	55-59	71.9%	1,336.25
	60-64	71.9%	1,486.25
Female	15-19	29.7%	595.51
	20-24	29.7%	708.15
	25-29	82.7%	841.86
	30-34	82.7%	972.20
	35-39	82.7%	1,066.3
	40-44	82.7%	1,105.07
	45-49	82.7%	1,113,78
	50-54	61.9%	1,152.38
	55-59	61.9%	1,164.06
	60-64	61.9%	1,203.67
